# Association Between Receipt of Overlapping Opioid and Benzodiazepine Prescriptions From Multiple Prescribers and Overdose Risk

**DOI:** 10.1001/jamanetworkopen.2021.20353

**Published:** 2021-08-10

**Authors:** Kao-Ping Chua, Chad M. Brummett, Sophia Ng, Amy S. B. Bohnert

**Affiliations:** 1 Department of Pediatrics, Susan B. Meister Child Health Evaluation and Research Center, University of Michigan, Ann Arbor; 2 Department of Health Management and Policy, University of Michigan School of Public Health, Ann Arbor; 3 Division of Pain Medicine, Department of Anesthesiology, University of Michigan Medical School, Ann Arbor; 4 Michigan Opioid Prescribing Engagement Network, Ann Arbor; 5 VA Center for Clinical Management Research, VA Ann Arbor Healthcare System, Ann Arbor, Michigan

## Abstract

**Importance:**

The receipt of overlapping opioid and benzodiazepine prescriptions is associated with increased overdose risk. It is unknown whether this increase in risk varies when overlapping prescriptions are written by multiple prescribers vs 1 prescriber.

**Objective:**

To evaluate the association between receipt of overlapping opioid and benzodiazepine prescriptions from multiple prescribers and overdose risk.

**Design, Setting, and Participants:**

This cohort study was conducted using 2017 to 2018 claims from the Optum deidentified Clinformatics Data Mart. Participants were patients with private insurance or Medicare Advantage aged 12 years or older with overlapping opioid and benzodiazepine prescriptions. Data were analyzed from March through November 2020.

**Exposures:**

For each patient, person-days on which opioid and benzodiazepine prescriptions overlapped were identified. The exposure was whether these prescriptions were written by multiple prescribers vs 1 prescriber.

**Main Outcomes and Measures:**

The outcome was a treated overdose, defined as the occurrence of 1 or more claims containing a diagnosis code for opioid or benzodiazepine poisoning on a person-day of opioid-benzodiazepine overlap. The association between exposure and outcome at the person-day level was estimated using logistic regression, controlling for opioid and benzodiazepine prescribing patterns, demographics, and comorbidities. The average marginal effect (AME) of the exposure, defined as the absolute difference in the probability of a treated overdose if all person-days of overlap involved prescriptions from multiple prescribers vs 1 prescriber, was calculated.

**Results:**

Among 529 053 patients, the mean (SD) age was 61.2 (15.6) years and 350 857 (66.3%) were female patients. Mean (SD) follow-up was 198.7 (249.8) days. During follow-up, overdose occurred on 1 or more person-days of opioid-benzodiazepine overlap for 2288 patients (0.4%, or 1 in 231 patients). There were 52 989 316 person-days of opioid-benzodiazepine overlap. Among 19 895 457 person-days (37.5%) involving prescriptions from multiple prescribers, there were 1390 overdoses (7.0 per 100 000 person-days), and among 33 093 859 person-days (62.5%) involving prescriptions from 1 prescriber, there were 1302 overdoses (3.9 per 100 000 person-days). Overdose risk was increased 1.8-fold (95% CI, 1.6-1.9) on person-days of overlap involving prescriptions from multiple prescribers vs 1 prescriber. The association between multiple prescribers and increased risk of overdose persisted in adjusted analyses (adjusted odds ratio, 1.20; 95% CI, 1.10-1.31; AME, 0.91 per 100 000 person-days of overlap; 95% CI, 0.46-1.37).

**Conclusions and Relevance:**

This study found that among patients already at increased risk of overdose owing to concurrent treatment with opioids and benzodiazepines, overdose risk was increased further when multiple prescribers were responsible for this treatment regimen compared with 1 prescriber. This increased risk was not fully accounted for by differences in prescribing patterns, demographics, or comorbidities. This finding suggests that other factors, such as poor care coordination, may be associated with the increase in risk.

## Key Points

**Question:**

Is overdose risk increased when overlapping opioid and benzodiazepine prescriptions are written by multiple prescribers vs 1 prescriber?

**Findings:**

In this cohort study of 529 053 patients with private insurance or Medicare Advantage, overdose risk was increased 1.8-fold when opioid-benzodiazepine overlap involved prescriptions from multiple prescribers vs 1 prescriber. This increase remained statistically significant after adjusting for prescribing patterns, demographics, and comorbidities.

**Meaning:**

This study found that observed factors did not fully account for the association between receipt of overlapping opioid and benzodiazepine prescriptions from multiple prescribers and overdose risk. This finding suggests that other factors, such as poor care coordination, may play a role.

## Teaser

This cohort study investigates the association of overlapping opioid and benzodiazepine prescriptions with overdose risk when prescribed by multiple vs single prescribers.

## Introduction

In each month of 2017, approximately one-fifth of US patients with dispensed opioid prescriptions had at least 1 day of overlapping opioid and benzodiazepine exposure.^1^ During the first half of 2018, 32.5% of US opioid-related overdose deaths involved benzodiazepines.^2^ The high prevalence of overlapping opioid and benzodiazepine prescriptions, coupled with the potential of this treatment regimen to increase overdose risk,^1,2,3,4,5,6^ highlights the importance of avoiding concurrent treatment with opioids and benzodiazepines when possible and mitigating overdose risk when not possible.

There are several reasons to suspect that the receipt of overlapping opioid and benzodiazepine prescriptions from multiple prescribers may be associated with increased overdose risk compared with the receipt of overlapping prescriptions from 1 prescriber. In 1 study^7^ of patients with overlapping opioid and benzodiazepine prescriptions, those receiving these prescriptions from multiple prescribers had opioid prescriptions with more days supplied, higher daily dosages, and longer periods of overlap with benzodiazepine prescriptions, prescribing patterns that are associated with increased overdose risk. Furthermore, the receipt of overlapping opioid and benzodiazepine prescriptions from multiple prescribers may be associated with poor care coordination, which is a potential risk factor associated with overdose.^8^ Despite these considerations, no study to our knowledge has evaluated the association between the receipt of overlapping opioid and benzodiazepine prescriptions from multiple prescribers and overdose risk. Closing this gap may determine whether patients receiving overlapping prescriptions from multiple prescribers warrant targeted overdose-prevention efforts.

Using national claims databases, we identified patients with private insurance or Medicare Advantage aged 12 years or older who had overlapping opioid and benzodiazepine prescriptions during 2017 to 2018. We assessed whether overdose risk differed when opioid-benzodiazepine overlap involved prescriptions from multiple prescribers vs 1 prescriber.

## Methods

Because data were deidentified, this cohort study was exempted from review by the institutional review board of the University of Michigan Medical School. The University of Michigan Medical School also determined that informed consent was not required because the data were deidentified. This study follows the Strengthening the Reporting of Observational Studies in Epidemiology (STROBE) reporting guideline for cohort studies.

### Data Sources

From March to November 2020, we conducted a retrospective cohort analysis using the 2017 to 2018 Optum deidentified Clinformatics Data Mart. This database contains medical and pharmacy claims from 17 million to 19 million patients with private insurance or Medicare Advantage from all 50 states. Data elements include demographics, month of death (but not cause of death), diagnosis codes, prescription information (eg, days supplied and quantity), and unique clinician identifiers constructed by Optum based on known information about clinicians and business intelligence rules. We used 2016 data as a look back for variables requiring a baseline period.

### Identification of Opioid-Benzodiazepine Overlap

We identified pharmacy claims for opioid and benzodiazepine prescriptions dispensed from 2017 to 2018 and the last quarter of 2016 (see eAppendix 1 in the Supplement for a list of medications). Opioids excluded opioid cough and cold medications. Following prior literature,^6,9^ we converted opioid and benzodiazepine pharmacy claims to person-days of exposure that would have occurred if patients took medications as prescribed. This exposure period began on the dispensing date and ended on the dispensing date plus days supplied minus 1 day. Person-days of opioid-benzodiazepine overlap were those on which concurrent exposure to opioids and benzodiazepines occurred.

### Sample

**Figure 1.**
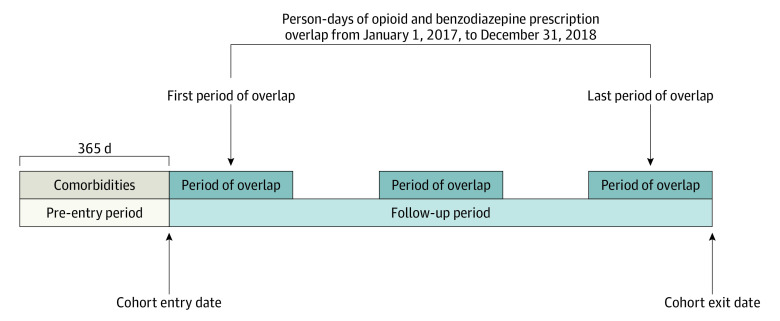
Study Design The cohort entry date is the first person-day of opioid-benzodiazepine overlap. The cohort exit date is depicted as the last person-day of opioid-benzodiazepine overlap during the study period. However, the cohort exit date could also be the first date of disenrollment from health insurance or the last date of the month of death (if applicable). Dark blue boxes indicate person-days of opioid-benzodiazepine overlap between cohort entry and exit.

We included patients aged 12 years or older who had 1 or more person-days of opioid-benzodiazepine overlap from January 1, 2017, to December 31, 2018 (ie, the study period). Prescriptions dispensed in the last quarter of 2016 could contribute to person-days of opioid-benzodiazepine overlap in early 2017. The cohort entry date was the first person-day of opioid-benzodiazepine overlap during the study period. The cohort exit date was the earliest of the following: last person-day of overlap during the study period, first date of disenrollment from health insurance, or last day of the month of death. Analyses included only person-days of overlap between cohort entry and exit. Figure 1 depicts the study design.

To capture baseline comorbidities, we required continuous enrollment 365 days prior to cohort entry. We excluded patients with 1 or more person-days of opioid-benzodiazepine overlap between cohort entry and exit that derived from opioid or benzodiazepine prescriptions with missing or invalid dosing data (defined as quantity <0, days supplied <0, or days supplied >90), patients with 1 or more person-days of overlap between cohort entry and exit that derived from opioid or benzodiazepine prescriptions for which the clinician identifier was missing or mapped to a nonprescriber, patients with missing demographic data, and patients residing in Puerto Rico. For patients who died during follow-up (eg, patients who filled opioid or benzodiazepine prescriptions shortly before death), we excluded person-days of opioid-benzodiazepine overlap after the month of death.

### Exposure

The exposure was an indicator that equaled 1 if the number of unique clinician identifiers across opioid and benzodiazepine prescriptions active on the person-day was 2 or more (ie, there were multiple prescribers). If 3 prescriptions were active on the person-day (eg, 2 opioid prescriptions and 1 benzodiazepine prescription), the exposure variable equaled 1 even if 1 prescriber accounted for 1 of the 2 opioid prescriptions and the benzodiazepine prescription, while another prescriber accounted for the other opioid prescription. In this example, opioid-benzodiazepine overlap still involved prescriptions from multiple prescribers: the opioid prescription from the latter prescriber and the benzodiazepine prescription from the former. Patients could contribute person-days of overlap involving multiple prescribers only, person-days of overlap involving one prescriber only, or a mixture.

### Outcome

The outcome was a treated overdose, defined as the occurrence of 1 or more medical claims containing a diagnosis code for opioid or benzodiazepine poisoning on a person-day of opioid-benzodiazepine overlap (see eTable 1 in the Supplement for a list of codes). We included opioid and benzodiazepine poisoning codes, given that clinicians may use either for overdoses among patients with concurrent opioid and benzodiazepine use. When overdose claims occurred on consecutive person-days (eg, a multiday hospitalization for overdose), we assigned the overdose event to the first person-day. We considered overdose claims separated by 2 or more days to represent distinct overdose events.^6,10^

### Covariates

We calculated daily opioid dosage in morphine milligram equivalents (MMEs), a standardized measure of opioid potency, by multiplying strength per dose, quantity, and published MME conversion factors, then dividing by days supplied (see eTable 2 in the Supplement for conversion factors used).^11,12^ When multiple opioid prescriptions were active on a person-day, we summed daily MMEs across prescriptions. Following prior literature, we categorized daily MMEs as 0 to 30 MMEs, 30 to 59 MMEs, 60 to 89 MMEs, 90 to 119 MMEs, and 120 MMEs or more.^6^ A 30-MME increment represents the difference between prescribing 1 vs 2 pills containing 5 mg hydrocodone every 4 hours.

We calculated daily benzodiazepine dosage in diazepam milligram equivalents (DMEs) using a similar approach as for daily MMEs, but we used DME conversion factors from previous studies.^13^ Following prior literature,^3^ we categorized daily DMEs as 0 to 10 DMEs, 11 to 20 DMEs, 21 to 30 DMEs, 31 to 40 DMEs, and 40 or more DMEs.

We created an indicator for use of extended-release, long-acting opioids, defined as having 1 or more active prescriptions for these medications on the person-day. We classified extended-release, long-acting opioids based on master form (eg, extended-release patch) or opioid type (for types with no short-acting form).

We created indicators for comorbidities, including cancer, mental health disorders, substance use disorders, and tobacco use. Additionally, we created a variable for number of Elixhauser comorbidity flags,^14^ excluding those for cancer, mental health disorders, and substance use disorders. We defined comorbidities based on the presence of 1 or more claims with a corresponding diagnosis code on or during the 365 days prior to cohort entry (see eAppendix 2 in the Supplement for code list).

We included demographic variables, including age, sex, US Census region, and payer type (ie, Medicare Advantage vs private). We created an indicator for the year during which the person-day occurred (ie, 2018 vs 2017). Except for comorbidities, covariates were time varying.

### Statistical Analysis

Using descriptive statistics, we assessed patient characteristics at cohort entry. To describe the prevalence of the exposure, we calculated the proportion of patients who had person-days with the exposure only, had person-days without the exposure only, or had a mixture. Moreover, we calculated the proportion of person-days of overlap with and without the exposure. Using taxonomy codes, we determined which prescriber types most frequently accounted for the opioid exposure and benzodiazepine exposure on person-days of overlap (eAppendix 3 in the Supplement).

We calculated the proportion of patients with 1 or more overdoses between cohort entry and exit, the rate of overdose per 100 000 person-days of opioid-benzodiazepine overlap, the unadjusted risk ratio for overdose among person-days of overlap with and without the exposure, and the prevalence of covariates among person-days of overlap with and without the exposure. In adjusted analyses, we evaluated the association between the exposure and outcome using logistic regression, controlling for covariates. Models employed standard errors clustered at the patient level. This approach is analogous to a discrete-time survival analysis assuming a constant hazard function.^15^ Models did not include higher-order terms for continuous covariates, given that they were not statistically significant. To improve interpretability, we calculated the average marginal effect (AME) of the exposure, representing the absolute difference in the probability of overdose if all person-days of overlap involved prescriptions from multiple prescribers vs 1 prescriber, holding covariates at their observed values.^16^

We used SAS statistical software version 9.4 (SAS Institute), Stata/MP statistical software version 16 (StataCorp), and R statistical software version 3.6.3 (R Project for Statistical Computing). We conducted 2-sided hypothesis tests with a significance level of α = .05.

We conducted several sensitivity analyses. The main analysis included only overdoses on person-days of opioid-benzodiazepine overlap, given that defining covariates, such as daily opioid dosage, would be difficult on person-days with no opioid exposure. To assess whether conclusions would change if we included overdoses that occurred shortly after periods of opioid-benzodiazepine overlap, we identified person-days associated with overdose that occurred within 7 days and 30 days of the last person-day of a continuous period of opioid-benzodiazepine overlap and that were not included in the main analysis. We calculated unadjusted overdose risk overall and by exposure, assuming these person-days had the same exposure status as the last person-day of the continuous period and assuming the number of person-days of overlap in the sample remained constant.

In additional sensitivity analyses, we excluded person-days of overlap involving prescriptions from pain medicine physicians (in case this indicator was a proxy for unobserved confounders, such as pain severity or differences in treatment practices), excluded person-days involving buprenorphine (owing to controversy over its MME conversion factor), and excluded patients with cancer or substance use disorders. To test sensitivity to regression specification, we controlled for number of days since the beginning of an episode of opioid-benzodiazepine overlap (thus allowing for time-varying risk), used a thin plate smoothing spline function rather than modeling daily MME and daily DME as categorical variables,^17^ and did not control for daily MME, daily DME, or use of extended-release, long-acting opioids, given that prescribing patterns may be on the causal pathway between the exposure and outcome. Finally, we modeled the exposure variable as categorical (ie, 1, 2, or ≥3 prescribers).

## Results

### Sample

**Figure 2.**
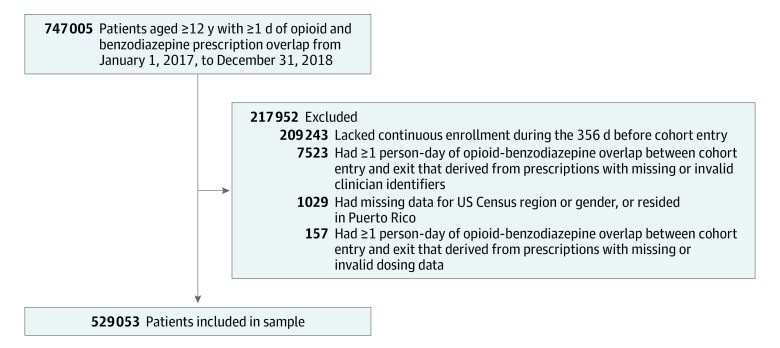
Sample Inclusion and Exclusion Criterion

Among 747 005 patients aged 12 years or older with 1 or more person-days of opioid-benzodiazepine overlap from January 1, 2017, to December 31, 2018, we excluded 207 952 patients (27.8%), leaving 529 053 patients (Figure 2). Table 1 displays sample demographic characteristics at cohort entry. The mean (SD) age was 61.2 (15.6) years, 350 857 (66.3%) were female patients, and 277 280 patients (52.4%) lived in the South. The mean (SD) duration of follow-up from cohort entry to exit was 198.7 (249.8) days.

**Table 1.** Patient Characteristics

Characteristic	No. (%) (N = 529 053)
Age group, y	
12-25	11 729 (2.2)
26-44	68 851 (13.0)
45-64	206 755 (38.1)
≥65	241 718 (45.7)
Sex	
Female patients	350 857 (66.3)
Male patients	178 196 (33.7)
US Census region	
Northeast	38 599 (7.3)
Midwest	104 014 (19.7)
South	277 280 (52.4)
West	109 160 (20.6)
Payer type	
Commercial	207 483 (39.2)
Medicare Advantage	321 570 (60.8)
Diagnosis	
Mental health disorder	
Yes	372 516 (70.4)
No	153 637 (29.6)
Substance use disorder	
Yes	57 604 (10.9)
No	471 449 (89.1)
Tobacco use	
Yes	122 696 (23.2)
No	406 357 (76.8)
Diagnosis of cancer	
Yes	128 012 (24.2)
No	401 041 (75.8)
Elixhauser comorbidity flags, No.^a^	
0-1	152 204 (28.8)
2-3	128 798 (24.4)
4-5	102 285 (19.3)
>5	145 766 (27.6)
Year of cohort entry	
2017	353 433 (66.8)
2018	175 620 (33.2)

^a^ Count excludes Elixhauser comorbidity flags related to mental health disorders, substance use disorders, and cancer.

### Exposure Prevalence

Among 529 053 patients, 147 081 patients (27.8%) had person-days of opioid-benzodiazepine overlap involving prescriptions from multiple prescribers only, 233 508 patients (44.1%) had person-days of overlap involving prescriptions from 1 prescriber only, and 148 464 patients (28.1%) had a mixture. There were 52 989 316 person-days of opioid-benzodiazepine overlap (mean [SD], 100.2 [163.3] days per patient). Of these, 19 895 457 person-days (37.5%) involved prescriptions from multiple prescribers, while 33 093 859 person-days (62.5%) involved prescriptions from 1 prescriber.

### Prescriber Types Accounting for the Exposure

Of 19 895 457 person-days of opioid-benzodiazepine overlap involving prescriptions from multiple prescribers, opioid prescriptions from pain medicine physicians were involved on 5 917 201 person-days (29.7%), compared with 3 765 569 person-days (18.9%) for internists and 3 041 792 person-days (15.3%) for family medicine physicians. Benzodiazepine prescriptions from family medicine physicians were involved on 5 706 768 person-days (28.7%), compared with 5 257 307 person-days (26.4%) for internists and 4 350 732 person-days (21.9%) for psychiatrists. Among 33 093 859 person-days of opioid-benzodiazepine overlap involving prescriptions from 1 prescriber, family medicine physicians accounted for 13 945 036 person-days (42.1%), compared with 11 045 193 person-days (33.4%) for internists and 2 009 865 person-days (6.1%) for nurse practitioners (Table 2).

**Table 2.** Prescribers Accounting for Prescriptions During Periods of Opioid-Benzodiazepine Overlap

With multiple prescribers				With 1 prescriber	
Prescriber accounting for active opioid prescriptions^a^	Person-days, No. (%) (n = 19 895 457)	Prescriber accounting for active benzodiazepine prescriptions	Person-days, No. (%) (n = 19 895 457)	Prescriber accounting for overlapping prescriptions	Person-days, No. (%) (n = 33 093 859)
Pain medicine physician	5 917 201 (29.7)	Family medicine physician	5 706 768 (28.7)	Family medicine physician	13 945 036 (42.1)
Internal medicine physician	765 569 (18.9)	Internal medicine physician	5 257 307 (26.4)	Internal medicine physician	11 045 193 (33.4)
Family medicine physician	3 041 792 (15.3)	Psychiatrist	4 350 732 (21.9)	Nurse practitioner	2 009 865 (6.1)
Nurse practitioner	2 015 089 (10.1)	Nurse practitioner	2 228 575 (11.2)	Pain medicine physician	1 168 566 (3.5)
Physical medicine and rehabilitation physician	1 450 180 (7.3)	Physician assistant	756 189 (3.8)	Physician assistant	947 124 (2.9)

^a^ The top 5 prescriber types are displayed.

### Overdose Rate

Among 529 053 patients in the sample, 2288 patients had 1 or more overdoses between cohort entry and exit (0.4%, or approximately 1 in 231) and 404 patients (0.08%) had multiple overdoses. Among 52 989 316 person-days of opioid-benzodiazepine overlap, there were 2692 treated overdoses (5.1 per 100 000 person-days), of which 157 overdoses (5.8%) occurred during the month of death. Among 19 895 457 person-days of opioid-benzodiazepine overlap involving prescriptions from multiple prescribers, there were 1390 overdoses (7.0 per 100 000 person-days). Among 33 093 859 person-days of opioid-benzodiazepine overlap involving prescriptions from 1 prescriber, there were 1302 overdoses (3.9 per 100 000 person-days). The unadjusted overdose risk was increased 1.8-fold (95% CI, 1.6-1.9) for the former compared with the latter.

### Prevalence of Covariates by Exposure

Table 3 displays the prevalence of covariates on person-days of opioid-benzodiazepine overlap involving prescriptions from multiple prescribers vs 1 prescriber. Person-days involving multiple prescribers, compared with those involving 1 prescriber, were more likely to have high-risk prescribing patterns, including daily MMEs of 90 or more (5 175 658 person-days [26.0%] vs 6 431 666 person-days [19.4%]) daily DMEs of 40 or more (2 657 762 person-days [13.4%] vs 3 157 074 person-days [9.5%]), and extended-release, long-acting opioid use (4 933 599 person-days [24.8%] vs 5 103 225 person-days [15.4%]), and were more likely to involve patients with mental health disorders (18 329 630 person-days [92.1%] vs 28 627 619 person-days [86.5%]) and substance use disorders (4 234 076 person-days [21.3%] vs 5 339 067 person-days [16.1%]).

**Table 3.** Covariate Prevalence by Exposure Status

Covariate	Person-days, No. (%)	
	Multiple prescribers (n = 19 895 457)	1 Prescriber (n = 33 093 859)
Daily opioid dosage category, MMEs		
<30	5 608 378 (28.2)	12 448 020 (37.6)
30-59	6 112 543 (30.7)	10 262 114 (31.0)
60-89	2 998 888 (15.1)	3 952 059 (11.9)
90-119	1 506 260 (7.6)	1 639 809 (5.0)
≥120	3 669 388 (18.4)	4 791 857 (14.5)
Daily benzodiazepine dosage category, DMEs		
≤10	6 911 465 (34.7)	13 334 184 (40.3)
11-20	5 309 602 (26.7)	9 144 673 (27.6)
21-30	2 903 447 (14.6)	4 613 289 (13.9)
31-40	2 113 181 (10.6)	2 844 639 (8.6)
≥40	2 657 762 (13.4)	3 157 074 (9.5)
Extended-release, long-acting opioid use		
Yes	4 933 599 (24.8)	5 103 225 (15.4)
No	14 961 858 (75.2)	27 990 634 (84.6)
Mental health disorder		
Yes	18 329 630 (92.1)	28 627 619 (86.5)
No	1 565 827 (7.9)	4 466 240 (13.5)
Substance use disorder		
Yes	4 234 076 (21.3)	5 339 067 (16.1)
No	15 661 381 (78.7)	27 754 792 (83.9)
Tobacco use		
Yes	7 343 136 (36.9)	11 351 163 (34.3)
No	12 552 321 (63.1)	21 742 696 (65.7)
Cancer		
Yes	5 800 407 (29.2)	8 624 142 (26.1)
No	14 095 050 (70.8)	24 469 717 (73.9)
Elixhauser comorbidity flags, No.^a^		
0-1	1 883 945 (9.5)	4 386 082 (13.3)
2-3	4 118 753 (20.7)	7 707 336 (23.3)
4-5	4 646 989 (23.4)	7 677 049 (23.2)
>5	9 245 770 (46.5)	13 323 392 (40.3)
Age group, y		
12-25	54 752 (0.3)	97 311 (0.3)
26-44	1 571 341 (7.9)	2 226 928 (6.7)
45-64	9 959 096 (50.1)	14 027 133 (42.4)
≥65	8 310 268 (41.8)	16 742 487 (50.6)
Sex		
Female patients	13 787 193 (69.3)	21 529 676 (65.1)
Male patients	6 108 264 (30.7)	11 564 183 (34.9)
US Census region		
Northeast	1 183 680 (5.9)	2 308 007 (7.0)
Midwest	3 502 449 (17.6)	5 987 635 (18.1)
South	11 882 102 (59.7)	18 394 483 (55.6)
West	3 327 226 (16.7)	6 403 734 (19.4)
Payer type		
Commercial	4 560 342 (22.9)	7 547 069 (22.8)
Medicare Advantage	15 335 115 (77.1)	25 546 790 (77.2)
Year in which person-day occurred		
2017	10 696 376 (53.8)	17 433 322 (52.7)
2018	9 199 081 (46.2)	15 660 537 (47.3)

Abbreviations: DMEs, diazepam milligram equivalents; MMEs, morphine milligram equivalents.

^a^ Count excludes Elixhauser comorbidity flags related to mental health disorders, substance use disorders, and cancer.

### Adjusted Analyses

The receipt of overlapping opioid-benzodiazepine prescriptions from multiple prescribers was associated with 1.20-fold (95% CI, 1.10-1.31) higher adjusted odds of overdose compared with the receipt of overlapping prescriptions from 1 prescriber. The adjusted rates of overdose if all vs no person-days involved prescriptions from multiple prescribers were 5.56 overdoses per 100 000 person-days of overlap and 4.65 overdoses per 100 000 person-days of overlap (AME of exposure, 0.91; 95% CI, 0.46-1.37). See eTable 3 in the Supplement for regression coefficients for covariates.

### Sensitivity Analyses

We identified 465 person-days associated with overdose that occurred within 7 days of the last person-day of a continuous period of opioid-benzodiazepine overlap and that were not included in the main analysis. If these 465 person-days had been included, the unadjusted risk difference between person-days of overlap involving prescriptions from multiple prescribers vs 1 prescriber would have been 3.6 overdoses per 100 000 person-days of overlap compared with 3.1 overdoses in the main analysis. We identified 728 person-days associated overdose that occurred within 30 days of the last person-day of a continuous period of opioid-benzodiazepine overlap and that were not included in the main analysis. If these 728 person-days had been included, the unadjusted risk difference between person-days of overlap involving prescriptions from multiple prescribers vs 1 prescriber would have been 4.1 overdoses per 100,000 person-days of overlap compared with 3.1 overdoses in the main analysis.

The positive association between exposure and outcome remained in all sensitivity analyses (eg, for the analysis excluding person-days of overlap involving prescriptions from a pain medicine physician, the AME was 0.87 [95% CI, 0.39-1.35]). When modeling the exposure as a categorical variable, the AME for 2 prescribers vs 1 prescriber was 0.83 (95% CI, 0.83-1.29), while the AME for 3 subscribers vs 1 prescriber was 2.57 (95% CI, 0.98-4.15) (eTable 4 in the Supplement).

## Discussion

In this national cohort study of more than 500 000 patients with private insurance or Medicare Advantage, the unadjusted risk of overdose was increased 1.8-fold when opioid-benzodiazepine overlap involved prescriptions from multiple prescribers vs 1 prescriber. After controlling for differences in prescribing patterns, demographic characteristics, and comorbidities, the adjusted odds of overdose were 1.2-fold higher when opioid-benzodiazepine overlap involved prescriptions from multiple prescribers. This estimate corresponds to approximately a 20% increase in the risk of overdose, as odds ratios and risk ratios are similar when outcomes are rare. These findings suggest that among patients already at increased risk for overdose owing to concurrent treatment with opioids and benzodiazepines, risk is increased further when multiple prescribers are responsible for this treatment regimen.

Consistent with prior work,^7^ person-days of opioid-benzodiazepine overlap involving prescriptions from multiple prescribers were more likely to be associated with high-risk prescribing patterns, including high daily opioid and benzodiazepine dosages. However, after adjusting for these differences in prescribing patterns, as well as differences in demographic characteristics and comorbidities, the positive association between receipt of overlapping opioid and benzodiazepine prescriptions from multiple prescribers and overdose risk remained. This finding does not imply that the association is causal, given that the involvement of multiple prescribers does not increase the ability of opioids and benzodiazepines to depress respiration. Rather, these findings imply that the association is driven by factors not measured directly in analyses.

Given the lack of clinical details in claims data, we cannot definitively identify these factors. However, we speculate that 1 such factor may be poor care coordination. For example, prescribers of opioids may be unaware that patients have active benzodiazepine prescriptions owing to differing systems for tracking prescription use. Opioid prescribers may therefore miss opportunities to implement strategies to prevent overdose, such as naloxone coprescribing. A 2019 study^8^ found that the dual receipt of opioid prescriptions covered by the Veterans Administration and by Medicare Part D was associated with fatal opioid overdose, potentially consistent with the notion that poor care coordination may be associated with increased overdose risk.

In our study, 1 of 231 patients experienced an overdose during follow-up. This finding suggests the importance of avoiding overlapping opioid and benzodiazepine prescriptions when possible, regardless of whether prescriptions are written by multiple prescribers or 1 prescriber. That said, the greater risk among patients receiving overlapping prescriptions from multiple prescribers suggests that targeted interventions may be warranted for these patients. For example, if electronic health record systems are linked to prescription drug–monitoring program databases, an alert to coprescribe naloxone could be created for clinicians who order opioid prescriptions for patients with an active benzodiazepine prescription from another prescriber (or the reverse). Our findings suggest that such interventions should focus on pain medicine physicians, internists, family medicine physicians, and psychiatrists, given that these clinicians were frequently involved on person-days of opioid-benzodiazepine overlap involving prescriptions from multiple prescribers.

### Limitations

This study has several limitations. First, as noted above, we cannot determine whether the demonstrated association is driven by poor care coordination, owing to data limitations. Second, it is unclear whether results generalize to patients without insurance or to patients covered by Medicaid or traditional Medicare. Third, 157 overdoses occurred during the month of death, but it was unclear whether deaths were due to overdose. Fourth, analyses likely undercounted the number of overdoses. Opioid and benzodiazepine poisoning codes have imperfect sensitivity for detecting overdose, although they are likely highly specific.^18^ Moreover, our database captured only treated overdoses resulting in claims. Perhaps most importantly, we counted only overdoses occurring during periods of opioid-benzodiazepine overlap. Our sensitivity analysis, however, suggested that including overdoses that occurred within 7 days and 30 days of the end of a period of opioid-benzodiazepine overlap would have resulted in a greater unadjusted risk difference than that found in the main analysis.

## Conclusions

This study found that the receipt of overlapping opioid and benzodiazepine prescriptions from multiple prescribers was associated with increased overdose risk compared with the receipt of overlapping prescriptions from 1 prescriber. This increased risk was not fully accounted for by differences in prescribing patterns, demographics, and comorbidities. Further studies are needed to determine the mechanism associated with this increased risk.

## Notes

Supplement. **eAppendix 1.** Opioids and Benzodiazepines Included in Analyses **eTable 1.** Diagnosis Codes Used to Define Opioid And Benzodiazepine Poisoning **eTable 2.** Morphine Milligram Equivalent Conversion Factors Used in Analyses **eAppendix 2.** Diagnosis Codes Used to Define Comorbidities **eAppendix 3.** Prescriber Types **eTable 3.** Adjusted Association Between Overlapping Opioid and Benzodiazepine Prescriptions From Multiple Prescribers and Overdose **eTable 4.** Sensitivity Analyses
