# Supplementary material for: Association Between Receipt of Overlapping Opioid and Benzodiazepine Prescriptions From Multiple Prescribers and Overdose Risk
Source: JAMA Netw Open. 2021 Aug 10;4(8):e2120353. doi: 10.1001/jamanetworkopen.2021.20353 (PMC8356065; doi:10.1001/jamanetworkopen.2021.20353)
Supplement: Supplement. — eAppendix 1. Opioids and Benzodiazepines Included in Analyses eTable 1. Diagnosis Codes Used to Define Opioid And Benzodiazepine Poisoning eTable 2. Morphine Milligram Equivalent Conversion Factors Used in Analyses eAppendix 2. Diagnosis Codes Used to Define Comorbidities eAppendix 3. Prescriber Types eTable 3. Adjusted Association Between Overlapping Opioid and Benzodiazepine Prescriptions From Multiple Prescribers and Overdose eTable 4. Sensitivity Analyses [file jamanetwopen-e2120353-s001.pdf]

Supplemental Online Content

Chua KP, Brummett CM, Ng S, Bohnert ASB. Association between receipt of overlapping opioid and benzodiazepine prescriptions from multiple prescribers and overdose risk. *JAMA Netw Open*. 2021;4(8):e2120353. doi:10.1001/jamanetworkopen.2021.20353

eAppendix 1. Opioids and Benzodiazepines Included in Analyses

eTable 1. Diagnosis Codes Used to Define Opioid And Benzodiazepine Poisoning

eTable 2. Morphine Milligram Equivalent Conversion Factors Used in Analyses

eAppendix 2. Diagnosis Codes Used to Define Comorbidities

eAppendix 3. Prescriber Types

eTable 3. Adjusted Association Between Overlapping Opioid and Benzodiazepine Prescriptions From Multiple Prescribers and Overdose

eTable 4. Sensitivity Analyses

This supplemental material has been provided by the authors to give readers additional information about their work.

© 2021 Chua KP et al. *JAMA Network Open.*

eAppendix 1. Opioids and Benzodiazepines Included in Analyses

Opioid analgesics

Opioid analgesics included buprenorphine, butorphanol, codeine, dihydrocodeine, fentanyl, hydrocodone, hydromorphone, levomethadyl, levorphanol, methadone, meperidine, morphine, opium, oxycodone, oxymorphone, pentazocine, propoxyphene, tapentadol, and tramadol. We excluded opioid cough-and-cold products.

Benzodiazepines

Benzodiazepines included alprazolam, bromazepam, broritzolam, chlordiazepoxide, clobazam, clonazepam, clorazepate, diazepam, ethyl loflazepate, estazolam, flunitrazepam, flurazepam, halazepam, lorazepam, medazepam, nitrazepam, nordazepam, oxazepam, prazepam, quazepam, temazepam, triazolam, triazulenone, and tofisopam. We excluded midazolam, which is uncommonly used in the outpatient setting outside of emergency departments (e.g., procedural sedation).

© 2021 Chua KP et al. *JAMA Network Open.*

eTable 1. Diagnosis Codes Used to Define Opioid And Benzodiazepine Poisoning

**ICD-10-CM code**	**Description**
T400X1- T400X4	Poisoning by opium
T402X1- T402X4	Poisoning by other opioids
T403X1- T403X4	Poisoning by methadone
T404X1- T404X4	Poisoning by other synthetic narcotics
T40601- T40604	Poisoning by unspecified narcotics
T40691- T40694	Poisoning by other narcotics
T424X1-T424X4	Poisoning by benzodiazepines

© 2021 Chua KP et al. *JAMA Network Open.*

eTable 2. Morphine Milligram Equivalent Conversion Factors Used in Analyses

**Type of Opioid** (strength units)	**MME Conversion Factor**
Buprenorphine film/tablet (mg)	30
Buprenorphine patch (mcg/hr)	12.6
Buprenorphine film (mcg)	0.03
Butorphanol (mg)	7
Codeine (mg)	0.15
Dihydrocodeine (mg)	0.25
Fentanyl buccal or SL tablets, or lozenge/troche (mcg)	0.13
Fentanyl film or oral spray (mcg)	0.18
Fentanyl nasal spray (mcg)	0.16
Fentanyl patch (mcg)	7.2
Hydrocodone (mg)	1
Hydromorphone (mg)	4
Levorphanol tartrate (mg)	11
Meperidine hydrochloride (mg)	0.1
Methadone (mg)	3
Morphine (mg)	1
Opium (mg)	1
Oxycodone (mg)	1.5
Oxymorphone (mg)	3
Pentazocine (mg)	0.37
Tapentadol (mg)	0.4
Tramadol (mg)	0.1

Source: https://www.cms.gov/Medicare/Prescription-Drug-Coverage/PrescriptionDrugCovContra/Downloads/Opioid-Morphine-EQ-Conversion-Factors-Aug-2017.pdf

© 2021 Chua KP et al. *JAMA Network Open.*

eAppendix 2. Diagnosis Codes Used to Define Comorbidities

Cancer

C00-D09, D37-D49 (not D3A, benign neuroendocrine tumors)

Mental health conditions

For both mental health conditions and substance use disorders, diagnosis codes were based on a slightly modified version of the Agency for Healthcare Research and Quality Clinical Classifications Software diagnosis code grouping algorithm.

ICD-10-CM code	Description
F01	Vascular dementia
F02	Dementia in other diseases classified elsewhere
F03	Unspecified dementia
F04	Amnestic disorder due to known physiological condition
F05	Delirium due to known physiological condition
F06	Other mental disorders due to known physiological condition
F07	Personality and behavioral disorders due to known physiological condition
F21	Schizotypal disorder
F22	Delusional disorders
F23	Brief psychotic disorder
F24	Shared psychotic disorder
F25	Schizoaffective disorders
F28	Other psychotic disorder not due to a substance or known physiological condition
F29	Unspecified psychosis not due to a substance or known physiological condition
F30	Manic episode
F31	Bipolar disorder
F32	Major depressive disorder, single episode
F33	Major depressive disorder, recurrent
F34	Persistent mood [affective] disorders
F39	Unspecified mood [affective] disorder
F40	Phobic anxiety disorders
F41	Other anxiety disorders
F42	Obsessive-compulsive disorder
F43	Reaction to severe stress, and adjustment disorders
F44	Dissociative and conversion disorders
F45	Somatoform disorders
F48	Other nonpsychotic mental disorders
F50	Eating disorders
F51	Sleep disorders not due to a substance or known physiological condition
F52	Sexual dysfunction not due to a substance or known physiological condition
F53	Puerperal psychosis
F54	Psychological and behavioral factors associated with disorders or diseases classified elsewhere
F59	Unspecified behavioral syndromes associated with physiological disturbances and physical factors
F60	Specific personality disorders
F63	Impulse disorders

© 2021 Chua KP et al. *JAMA Network Open.*

F64	Gender identity disorders
F65	Paraphilias
F66	Other sexual disorders
F68	Other disorders of adult personality and behavior
F69	Unspecified disorder of adult personality and behavior
F70	Mild intellectual disabilities
F71	Moderate intellectual disabilities
F72	Severe intellectual disabilities
F73	Profound intellectual disabilities
F78	Other intellectual disabilities
F79	Unspecified intellectual disabilities
F80	Specific developmental disorders of speech and language
F81	Specific developmental disorders of scholastic skills
F82	Specific developmental disorder of motor function
F84	Pervasive developmental disorders
F88	Other disorders of psychological development
F89	Unspecified disorder of psychological development
F90	Attention-deficit hyperactivity disorders
F91	Conduct disorders
F93	Emotional disorders with onset specific to childhood
F94	Disorders of social functioning with onset specific to childhood and adolescence
F95	Tic disorder
F98	Other behavioral and emotional disorders with onset usually occurring in childhood and adolescence
F99	Mental disorder, not otherwise specified
O906	Postpartum mood disturbance
R37	Sexual dysfunction, unspecified
R45	Symptoms and signs involving emotional state
R46	Symptoms and signs involving appearance and behavior
T1491	Suicide attempt
T36-T39, T405X2, T407X2, T408X2, T41-T71 and X71-X83 (intentional self-harm codes)	Intentional self-harm
Z6281	Personal history of abuse in childhood
Z690	Encounter for mental health services for child abuse problems
Z6902	Encounter for mental health services for non-parental child abuse
Z691	Encounter for mental health services for spousal or partner abuse problems
Z698	Encounter for mental health services for victim or perpetrator of other abuse
Z7281	Antisocial behavior
Z865	Personal history of mental and behavioral disorders
Z914	Personal history of psychological trauma, not elsewhere classified
Z915	Personal history of self-harm

Substance use disorders

© 2021 Chua KP et al. *JAMA Network Open.*

ICD-10-CM code	Description
F10-F16; F18-F19 (except for codes for uncomplicated use and uncomplicated dependence)	Disorders related to alcohol, opioids, cannabis, sedative/hypnotic/anxiolytics, cocaine, other stimulants, hallucinogens, inhalants, other psychoactive substances
G312	Abuse of non-psychoactive substances
G312	Degeneration of nervous system due to alcohol
G621	Alcoholic polyneuropathy
I426	Alcoholic cardiomyopathy
K2920	Alcoholic gastritis without bleeding
K2921	Alcoholic gastritis with bleeding
K700	Alcoholic fatty liver
K7010	Alcoholic hepatitis without ascites
K7011	Alcoholic hepatitis with ascites
K702	Alcoholic fibrosis and sclerosis of liver
K7030	Alcoholic cirrhosis of liver without ascites
K7031	Alcoholic cirrhosis of liver with ascites
K7040	Alcoholic hepatic failure without coma
K7041	Alcoholic hepatic failure with coma
K709	Alcoholic liver disease, unspecified
O354XX0	Maternal care for (suspected) damage to fetus from alcohol, not applicable or unspecified
O354XX1	Maternal care for (suspected) damage to fetus from alcohol, fetus 1
O354XX2	Maternal care for (suspected) damage to fetus from alcohol, fetus 2
O354XX3	Maternal care for (suspected) damage to fetus from alcohol, fetus 3
O354XX4	Maternal care for (suspected) damage to fetus from alcohol, fetus 4
O354XX5	Maternal care for (suspected) damage to fetus from alcohol, fetus 5
O354XX9	Maternal care for (suspected) damage to fetus from alcohol, other fetus
O355XX0	Maternal care for (suspected) damage to fetus by drugs, not applicable or unspecified
O355XX1	Maternal care for (suspected) damage to fetus by drugs, fetus 1
O355XX2	Maternal care for (suspected) damage to fetus by drugs, fetus 2
O355XX3	Maternal care for (suspected) damage to fetus by drugs, fetus 3
O355XX4	Maternal care for (suspected) damage to fetus by drugs, fetus 4
O355XX5	Maternal care for (suspected) damage to fetus by drugs, fetus 5
O355XX9	Maternal care for (suspected) damage to fetus by drugs, other fetus
O99310	Alcohol use complicating pregnancy, unspecified trimester
O99311	Alcohol use complicating pregnancy, first trimester
O99312	Alcohol use complicating pregnancy, second trimester
O99313	Alcohol use complicating pregnancy, third trimester
O99314	Alcohol use complicating childbirth
O99315	Alcohol use complicating the puerperium
O99320	Drug use complicating pregnancy, unspecified trimester
O99321	Drug use complicating pregnancy, first trimester
O99322	Drug use complicating pregnancy, second trimester
O99323	Drug use complicating pregnancy, third trimester
O99324	Drug use complicating childbirth

© 2021 Chua KP et al. *JAMA Network Open.*

O99325	Drug use complicating the puerperium
T400X1D	Poisoning by opium, accidental (unintentional), subsequent encounter
T400X1S	Poisoning by opium, accidental (unintentional), sequela
T400X2A	Poisoning by opium, intentional self-harm, initial encounter
T400X2D	Poisoning by opium, intentional self-harm, subsequent encounter
T400X2S	Poisoning by opium, intentional self-harm, sequela
T400X3A	Poisoning by opium, assault, initial encounter
T400X3D	Poisoning by opium, assault, subsequent encounter
T400X3S	Poisoning by opium, assault, sequela
T400X4A	Poisoning by opium, undetermined, initial encounter
T400X4D	Poisoning by opium, undetermined, subsequent encounter
T400X4S	Poisoning by opium, undetermined, sequela
T400X5A	Adverse effect of opium, initial encounter
T400X5D	Adverse effect of opium, subsequent encounter
T400X5S	Adverse effect of opium, sequela
T401X1A	Poisoning by heroin, accidental (unintentional), initial encounter
T401X1D	Poisoning by heroin, accidental (unintentional), subsequent encounter
T401X1S	Poisoning by heroin, accidental (unintentional), sequela
T401X2A	Poisoning by heroin, intentional self-harm, initial encounter
T401X2D	Poisoning by heroin, intentional self-harm, subsequent encounter
T401X2S	Poisoning by heroin, intentional self-harm, sequela
T401X3A	Poisoning by heroin, assault, initial encounter
T401X3D	Poisoning by heroin, assault, subsequent encounter
T401X3S	Poisoning by heroin, assault, sequela
T401X4A	Poisoning by heroin, undetermined, initial encounter
T401X4D	Poisoning by heroin, undetermined, subsequent encounter
T401X4S	Poisoning by heroin, undetermined, sequela
T402X1A	Poisoning by other opioids, accidental (unintentional), initial encounter
T402X1D	Poisoning by other opioids, accidental (unintentional), subsequent encounter
T402X1S	Poisoning by other opioids, accidental (unintentional), sequela
T402X2A	Poisoning by other opioids, intentional self-harm, initial encounter
T402X2D	Poisoning by other opioids, intentional self-harm, subsequent encounter
T402X2S	Poisoning by other opioids, intentional self-harm, sequela
T402X3A	Poisoning by other opioids, assault, initial encounter
T402X3D	Poisoning by other opioids, assault, subsequent encounter
T402X3S	Poisoning by other opioids, assault, sequela
T402X4A	Poisoning by other opioids, undetermined, initial encounter
T402X4D	Poisoning by other opioids, undetermined, subsequent encounter
T402X4S	Poisoning by other opioids, undetermined, sequela
T402X5A	Adverse effect of other opioids, initial encounter
T402X5D	Adverse effect of other opioids, subsequent encounter
T402X5S	Adverse effect of other opioids, sequela
T403X1A	Poisoning by methadone, accidental (unintentional), initial encounter
T403X1D	Poisoning by methadone, accidental (unintentional), subsequent encounter
T403X1S	Poisoning by methadone, accidental (unintentional), sequela

© 2021 Chua KP et al. *JAMA Network Open.*

T403X2A	Poisoning by methadone, intentional self-harm, initial encounter
T403X2D	Poisoning by methadone, intentional self-harm, subsequent encounter
T403X2S	Poisoning by methadone, intentional self-harm, sequela
T403X3A	Poisoning by methadone, assault, initial encounter
T403X3D	Poisoning by methadone, assault, subsequent encounter
T403X3S	Poisoning by methadone, assault, sequela
T403X4A	Poisoning by methadone, undetermined, initial encounter
T403X4D	Poisoning by methadone, undetermined, subsequent encounter
T403X4S	Poisoning by methadone, undetermined, sequela
T403X5A	Adverse effect of methadone, initial encounter
T403X5D	Adverse effect of methadone, subsequent encounter
T403X5S	Adverse effect of methadone, sequela
T404X1A	Poisoning by other synthetic narcotics, accidental (unintentional), initial encounter
T404X1D	Poisoning by other synthetic narcotics, accidental (unintentional), subsequent encounter
T404X1S	Poisoning by other synthetic narcotics, accidental (unintentional), sequela
T404X2A	Poisoning by other synthetic narcotics, intentional self-harm, initial encounter
T404X2D	Poisoning by other synthetic narcotics, intentional self-harm, subsequent encounter
T404X2S	Poisoning by other synthetic narcotics, intentional self-harm, sequela
T404X3A	Poisoning by other synthetic narcotics, assault, initial encounter
T404X3D	Poisoning by other synthetic narcotics, assault, subsequent encounter
T404X3S	Poisoning by other synthetic narcotics, assault, sequela
T404X4A	Poisoning by other synthetic narcotics, undetermined, initial encounter
T404X4D	Poisoning by other synthetic narcotics, undetermined, subsequent encounter
T404X4S	Poisoning by other synthetic narcotics, undetermined, sequela
T404X5A	Adverse effect of other synthetic narcotics, initial encounter
T404X5D	Adverse effect of other synthetic narcotics, subsequent encounter
T404X5S	Adverse effect of other synthetic narcotics, sequela
T405X1A	Poisoning by cocaine, accidental (unintentional), initial encounter
T405X1D	Poisoning by cocaine, accidental (unintentional), subsequent encounter
T405X1S	Poisoning by cocaine, accidental (unintentional), sequela
T405X2A	Poisoning by cocaine, intentional self-harm, initial encounter
T405X2D	Poisoning by cocaine, intentional self-harm, subsequent encounter
T405X2S	Poisoning by cocaine, intentional self-harm, sequela
T405X3A	Poisoning by cocaine, assault, initial encounter
T405X3D	Poisoning by cocaine, assault, subsequent encounter
T405X3S	Poisoning by cocaine, assault, sequela
T405X4A	Poisoning by cocaine, undetermined, initial encounter
T405X4D	Poisoning by cocaine, undetermined, subsequent encounter
T405X4S	Poisoning by cocaine, undetermined, sequela
T405X5A	Adverse effect of cocaine, initial encounter
T405X5D	Adverse effect of cocaine, subsequent encounter
T405X5S	Adverse effect of cocaine, sequela
T40601A	Poisoning by unspecified narcotics, accidental (unintentional), initial encounter
T40601D	Poisoning by unspecified narcotics, accidental (unintentional), subsequent encounter
T40601S	Poisoning by unspecified narcotics, accidental (unintentional), sequela

© 2021 Chua KP et al. *JAMA Network Open.*

T40602A	Poisoning by unspecified narcotics, intentional self-harm, initial encounter
T40602D	Poisoning by unspecified narcotics, intentional self-harm, subsequent encounter
T40602S	Poisoning by unspecified narcotics, intentional self-harm, sequela
T40603A	Poisoning by unspecified narcotics, assault, initial encounter
T40603D	Poisoning by unspecified narcotics, assault, subsequent encounter
T40603S	Poisoning by unspecified narcotics, assault, sequela
T40604A	Poisoning by unspecified narcotics, undetermined, initial encounter
T40604D	Poisoning by unspecified narcotics, undetermined, subsequent encounter
T40604S	Poisoning by unspecified narcotics, undetermined, sequela
T40605A	Adverse effect of unspecified narcotics, initial encounter
T40605D	Adverse effect of unspecified narcotics, subsequent encounter
T40605S	Adverse effect of unspecified narcotics, sequela
T40691A	Poisoning by other narcotics, accidental (unintentional), initial encounter
T40691D	Poisoning by other narcotics, accidental (unintentional), subsequent encounter
T40691S	Poisoning by other narcotics, accidental (unintentional), sequela
T40692A	Poisoning by other narcotics, intentional self-harm, initial encounter
T40692D	Poisoning by other narcotics, intentional self-harm, subsequent encounter
T40692S	Poisoning by other narcotics, intentional self-harm, sequela
T40693A	Poisoning by other narcotics, assault, initial encounter
T40693D	Poisoning by other narcotics, assault, subsequent encounter
T40693S	Poisoning by other narcotics, assault, sequela
T40694A	Poisoning by other narcotics, undetermined, initial encounter
T40694D	Poisoning by other narcotics, undetermined, subsequent encounter
T40694S	Poisoning by other narcotics, undetermined, sequela
T40695A	Adverse effect of other narcotics, initial encounter
T40695D	Adverse effect of other narcotics, subsequent encounter
T40695S	Adverse effect of other narcotics, sequela
T407X1A	Poisoning by cannabis (derivatives), accidental (unintentional), initial encounter
T407X1D	Poisoning by cannabis (derivatives), accidental (unintentional), subsequent encounter
T407X1S	Poisoning by cannabis (derivatives), accidental (unintentional), sequela
T407X2A	Poisoning by cannabis (derivatives), intentional self-harm, initial encounter
T407X2D	Poisoning by cannabis (derivatives), intentional self-harm, subsequent encounter
T407X2S	Poisoning by cannabis (derivatives), intentional self-harm, sequela
T407X3A	Poisoning by cannabis (derivatives), assault, initial encounter
T407X3D	Poisoning by cannabis (derivatives), assault, subsequent encounter
T407X3S	Poisoning by cannabis (derivatives), assault, sequela
T407X4A	Poisoning by cannabis (derivatives), undetermined, initial encounter
T407X4D	Poisoning by cannabis (derivatives), undetermined, subsequent encounter
T407X4S	Poisoning by cannabis (derivatives), undetermined, sequela
T407X5A	Adverse effect of cannabis (derivatives), initial encounter
T407X5D	Adverse effect of cannabis (derivatives), subsequent encounter
T407X5S	Adverse effect of cannabis (derivatives), sequela
T408X1A	Poisoning by lysergide [LSD], accidental (unintentional), initial encounter
T408X1D	Poisoning by lysergide [LSD], accidental (unintentional), subsequent encounter
T408X1S	Poisoning by lysergide [LSD], accidental (unintentional), sequela

© 2021 Chua KP et al. *JAMA Network Open.*

T408X2A	Poisoning by lysergide [LSD], intentional self-harm, initial encounter
T408X2D	Poisoning by lysergide [LSD], intentional self-harm, subsequent encounter
T408X2S	Poisoning by lysergide [LSD], intentional self-harm, sequela
T408X3A	Poisoning by lysergide [LSD], assault, initial encounter
T408X3D	Poisoning by lysergide [LSD], assault, subsequent encounter
T408X3S	Poisoning by lysergide [LSD], assault, sequela
T408X4A	Poisoning by lysergide [LSD], undetermined, initial encounter
T408X4D	Poisoning by lysergide [LSD], undetermined, subsequent encounter
T408X4S	Poisoning by lysergide [LSD], undetermined, sequela
T40901A	Poisoning by unspecified psychodysleptics [hallucinogens], accidental (unintentional), initial encounter
T40901D	Poisoning by unspecified psychodysleptics [hallucinogens], accidental (unintentional), subsequent encounter
T40901S	Poisoning by unspecified psychodysleptics [hallucinogens], accidental (unintentional), sequela
T40902A	Poisoning by unspecified psychodysleptics [hallucinogens], intentional self-harm, initial encounter
T40902D	Poisoning by unspecified psychodysleptics [hallucinogens], intentional self-harm, subsequent encounter
T40902S	Poisoning by unspecified psychodysleptics [hallucinogens], intentional self-harm, sequela
T40903A	Poisoning by unspecified psychodysleptics [hallucinogens], assault, initial encounter
T40903D	Poisoning by unspecified psychodysleptics [hallucinogens], assault, subsequent encounter
T40903S	Poisoning by unspecified psychodysleptics [hallucinogens], assault, sequela
T40904A	Poisoning by unspecified psychodysleptics [hallucinogens], undetermined, initial encounter
T40904D	Poisoning by unspecified psychodysleptics [hallucinogens], undetermined, subsequent encounter
T40904S	Poisoning by unspecified psychodysleptics [hallucinogens], undetermined, sequela
T40905A	Adverse effect of unspecified psychodysleptics [hallucinogens], initial encounter
T40905D	Adverse effect of unspecified psychodysleptics [hallucinogens], subsequent encounter
T40905S	Adverse effect of unspecified psychodysleptics [hallucinogens], sequela
T40991A	Poisoning by other psychodysleptics [hallucinogens], accidental (unintentional), initial encounter
T40991D	Poisoning by other psychodysleptics [hallucinogens], accidental (unintentional), subsequent encounter
T40991S	Poisoning by other psychodysleptics [hallucinogens], accidental (unintentional), sequela
T40992A	Poisoning by other psychodysleptics [hallucinogens], intentional self-harm, initial encounter
T40992D	Poisoning by other psychodysleptics [hallucinogens], intentional self-harm, subsequent encounter
T40992S	Poisoning by other psychodysleptics [hallucinogens], intentional self-harm, sequela
T40993A	Poisoning by other psychodysleptics [hallucinogens], assault, initial encounter
T40993D	Poisoning by other psychodysleptics [hallucinogens], assault, subsequent encounter
T40993S	Poisoning by other psychodysleptics [hallucinogens], assault, sequela
T40994A	Poisoning by other psychodysleptics [hallucinogens], undetermined, initial encounter
T40994D	Poisoning by other psychodysleptics [hallucinogens], undetermined, subsequent encounter
T40994S	Poisoning by other psychodysleptics [hallucinogens], undetermined, sequela
T40995A	Adverse effect of other psychodysleptics [hallucinogens], initial encounter
T40995D	Adverse effect of other psychodysleptics [hallucinogens], subsequent encounter
T40995S	Adverse effect of other psychodysleptics [hallucinogens], sequela

© 2021 Chua KP et al. *JAMA Network Open.*

T410X1A	Poisoning by inhaled anesthetics, accidental (unintentional), initial encounter
T410X1D	Poisoning by inhaled anesthetics, accidental (unintentional), subsequent encounter
T410X1S	Poisoning by inhaled anesthetics, accidental (unintentional), sequela
T410X2A	Poisoning by inhaled anesthetics, intentional self-harm, initial encounter
T410X2D	Poisoning by inhaled anesthetics, intentional self-harm, subsequent encounter
T410X2S	Poisoning by inhaled anesthetics, intentional self-harm, sequela
T410X3A	Poisoning by inhaled anesthetics, assault, initial encounter
T410X3D	Poisoning by inhaled anesthetics, assault, subsequent encounter
T410X3S	Poisoning by inhaled anesthetics, assault, sequela
T410X4A	Poisoning by inhaled anesthetics, undetermined, initial encounter
T410X4D	Poisoning by inhaled anesthetics, undetermined, subsequent encounter
T410X4S	Poisoning by inhaled anesthetics, undetermined, sequela
T410X5A	Adverse effect of inhaled anesthetics, initial encounter
T410X5D	Adverse effect of inhaled anesthetics, subsequent encounter
T410X5S	Adverse effect of inhaled anesthetics, sequela
T411X2S	Poisoning by intravenous anesthetics, intentional self-harm, sequela
T41202S	Poisoning by unspecified general anesthetics, intentional self-harm, sequela
T41292S	Poisoning by other general anesthetics, intentional self-harm, sequela
T413X2S	Poisoning by local anesthetics, intentional self-harm, sequela
T4142XS	Poisoning by unspecified anesthetic, intentional self-harm, sequela
T415X2S	Poisoning by therapeutic gases, intentional self-harm, sequela
T420X2S	Poisoning by hydantoin derivatives, intentional self-harm, sequela
T422X2S	Poisoning by succinimides and oxazolidinediones, intentional self-harm, sequela
T423X2S	Poisoning by barbiturates, intentional self-harm, sequela
T424X2S	Poisoning by benzodiazepines, intentional self-harm, sequela
T425X2S	Poisoning by mixed antiepileptics, intentional self-harm, sequela
T426X1A	Poisoning by other antiepileptic and sedative-hypnotic drugs, accidental (unintentional), initial encounter
T426X1D	Poisoning by other antiepileptic and sedative-hypnotic drugs, accidental (unintentional), subsequent encounter
T426X1S	Poisoning by other antiepileptic and sedative-hypnotic drugs, accidental (unintentional), sequela
T426X2A	Poisoning by other antiepileptic and sedative-hypnotic drugs, intentional self-harm, initial encounter
T426X2D	Poisoning by other antiepileptic and sedative-hypnotic drugs, intentional self-harm, subsequent encounter
T426X2S	Poisoning by other antiepileptic and sedative-hypnotic drugs, intentional self-harm, sequela
T426X3A	Poisoning by other antiepileptic and sedative-hypnotic drugs, assault, initial encounter
T426X3D	Poisoning by other antiepileptic and sedative-hypnotic drugs, assault, subsequent encounter
T426X3S	Poisoning by other antiepileptic and sedative-hypnotic drugs, assault, sequela
T426X4A	Poisoning by other antiepileptic and sedative-hypnotic drugs, undetermined, initial encounter
T426X4D	Poisoning by other antiepileptic and sedative-hypnotic drugs, undetermined, subsequent encounter
T426X4S	Poisoning by other antiepileptic and sedative-hypnotic drugs, undetermined, sequela
T426X5A	Adverse effect of other antiepileptic and sedative-hypnotic drugs, initial encounter
T426X5D	Adverse effect of other antiepileptic and sedative-hypnotic drugs, subsequent encounter

© 2021 Chua KP et al. *JAMA Network Open.*

T426X5S	Adverse effect of other antiepileptic and sedative-hypnotic drugs, sequela
T4271XA	Poisoning by unspecified antiepileptic and sedative-hypnotic drugs, accidental (unintentional), initial encounter
T4271XD	Poisoning by unspecified antiepileptic and sedative-hypnotic drugs, accidental (unintentional), subsequent encounter
T4271XS	Poisoning by unspecified antiepileptic and sedative-hypnotic drugs, accidental (unintentional), sequela
T4272XA	Poisoning by unspecified antiepileptic and sedative-hypnotic drugs, intentional self-harm, initial encounter
T4272XD	Poisoning by unspecified antiepileptic and sedative-hypnotic drugs, intentional self-harm, subsequent encounter
T4272XS	Poisoning by unspecified antiepileptic and sedative-hypnotic drugs, intentional self-harm, sequela
T4273XA	Poisoning by unspecified antiepileptic and sedative-hypnotic drugs, assault, initial encounter
T4273XD	Poisoning by unspecified antiepileptic and sedative-hypnotic drugs, assault, subsequent encounter
T4273XS	Poisoning by unspecified antiepileptic and sedative-hypnotic drugs, assault, sequela
T4274XA	Poisoning by unspecified antiepileptic and sedative-hypnotic drugs, undetermined, initial encounter
T4274XD	Poisoning by unspecified antiepileptic and sedative-hypnotic drugs, undetermined, subsequent encounter
T4274XS	Poisoning by unspecified antiepileptic and sedative-hypnotic drugs, undetermined, sequela
T4275XA	Adverse effect of unspecified antiepileptic and sedative-hypnotic drugs, initial encounter
T4275XD	Adverse effect of unspecified antiepileptic and sedative-hypnotic drugs, subsequent encounter
T4275XS	Adverse effect of unspecified antiepileptic and sedative-hypnotic drugs, sequela
T428X2S	Poisoning by antiparkinsonism drugs and other central muscle-tone depressants, intentional self-harm, sequela
T43012S	Poisoning by tricyclic antidepressants, intentional self-harm, sequela
T43022S	Poisoning by tetracyclic antidepressants, intentional self-harm, sequela
T431X2S	Poisoning by monoamine-oxidase-inhibitor antidepressants, intentional self-harm, sequela
T43202S	Poisoning by unspecified antidepressants, intentional self-harm, sequela
T43205S	Adverse effect of unspecified antidepressants, sequela
T43212S	Poisoning by selective serotonin and norepinephrine reuptake inhibitors, intentional self-harm, sequela
T43222S	Poisoning by selective serotonin reuptake inhibitors, intentional self-harm, sequela
T43292S	Poisoning by other antidepressants, intentional self-harm, sequela
T433X2S	Poisoning by phenothiazine antipsychotics and neuroleptics, intentional self-harm, sequela
T434X2S	Poisoning by butyrophenone and thiothixene neuroleptics, intentional self-harm, sequela
T43502S	Poisoning by unspecified antipsychotics and neuroleptics, intentional self-harm, sequela
T43592S	Poisoning by other antipsychotics and neuroleptics, intentional self-harm, sequela
T43601A	Poisoning by unspecified psychostimulants, accidental (unintentional), initial encounter
T43601D	Poisoning by unspecified psychostimulants, accidental (unintentional), subsequent encounter
T43601S	Poisoning by unspecified psychostimulants, accidental (unintentional), sequela
T43602A	Poisoning by unspecified psychostimulants, intentional self-harm, initial encounter
T43602D	Poisoning by unspecified psychostimulants, intentional self-harm, subsequent encounter
T43602S	Poisoning by unspecified psychostimulants, intentional self-harm, sequela
T43603A	Poisoning by unspecified psychostimulants, assault, initial encounter
T43603D	Poisoning by unspecified psychostimulants, assault, subsequent encounter
T43603S	Poisoning by unspecified psychostimulants, assault, sequela

© 2021 Chua KP et al. *JAMA Network Open.*

T43604A	Poisoning by unspecified psychostimulants, undetermined, initial encounter
T43604D	Poisoning by unspecified psychostimulants, undetermined, subsequent encounter
T43604S	Poisoning by unspecified psychostimulants, undetermined, sequela
T43605A	Adverse effect of unspecified psychostimulants, initial encounter
T43605D	Adverse effect of unspecified psychostimulants, subsequent encounter
T43605S	Adverse effect of unspecified psychostimulants, sequela
T43611A	Poisoning by caffeine, accidental (unintentional), initial encounter
T43611D	Poisoning by caffeine, accidental (unintentional), subsequent encounter
T43611S	Poisoning by caffeine, accidental (unintentional), sequela
T43612A	Poisoning by caffeine, intentional self-harm, initial encounter
T43612D	Poisoning by caffeine, intentional self-harm, subsequent encounter
T43612S	Poisoning by caffeine, intentional self-harm, sequela
T43613A	Poisoning by caffeine, assault, initial encounter
T43613D	Poisoning by caffeine, assault, subsequent encounter
T43613S	Poisoning by caffeine, assault, sequela
T43614A	Poisoning by caffeine, undetermined, initial encounter
T43614D	Poisoning by caffeine, undetermined, subsequent encounter
T43614S	Poisoning by caffeine, undetermined, sequela
T43615A	Adverse effect of caffeine, initial encounter
T43615D	Adverse effect of caffeine, subsequent encounter
T43615S	Adverse effect of caffeine, sequela
T43621A	Poisoning by amphetamines, accidental (unintentional), initial encounter
T43621D	Poisoning by amphetamines, accidental (unintentional), subsequent encounter
T43621S	Poisoning by amphetamines, accidental (unintentional), sequela
T43622A	Poisoning by amphetamines, intentional self-harm, initial encounter
T43622D	Poisoning by amphetamines, intentional self-harm, subsequent encounter
T43622S	Poisoning by amphetamines, intentional self-harm, sequela
T43623A	Poisoning by amphetamines, assault, initial encounter
T43623D	Poisoning by amphetamines, assault, subsequent encounter
T43623S	Poisoning by amphetamines, assault, sequela
T43624A	Poisoning by amphetamines, undetermined, initial encounter
T43624D	Poisoning by amphetamines, undetermined, subsequent encounter
T43624S	Poisoning by amphetamines, undetermined, sequela
T43625A	Adverse effect of amphetamines, initial encounter
T43625D	Adverse effect of amphetamines, subsequent encounter
T43625S	Adverse effect of amphetamines, sequela
T43631A	Poisoning by methylphenidate, accidental (unintentional), initial encounter
T43631D	Poisoning by methylphenidate, accidental (unintentional), subsequent encounter
T43631S	Poisoning by methylphenidate, accidental (unintentional), sequela
T43632A	Poisoning by methylphenidate, intentional self-harm, initial encounter
T43632D	Poisoning by methylphenidate, intentional self-harm, subsequent encounter
T43632S	Poisoning by methylphenidate, intentional self-harm, sequela
T43633A	Poisoning by methylphenidate, assault, initial encounter
T43633D	Poisoning by methylphenidate, assault, subsequent encounter
T43633S	Poisoning by methylphenidate, assault, sequela

T43634A	Poisoning by methylphenidate, undetermined, initial encounter
T43634D	Poisoning by methylphenidate, undetermined, subsequent encounter
T43634S	Poisoning by methylphenidate, undetermined, sequela
T43635A	Adverse effect of methylphenidate, initial encounter
T43635D	Adverse effect of methylphenidate, subsequent encounter
T43635S	Adverse effect of methylphenidate, sequela
T43641A	Poisoning by ecstasy, accidental (unintentional), initial encounter
T43641D	Poisoning by ecstasy, accidental (unintentional), subsequent encounter
T43641S	Poisoning by ecstasy, accidental (unintentional), sequela
T43642A	Poisoning by ecstasy, intentional self-harm, initial encounter
T43642D	Poisoning by ecstasy, intentional self-harm, subsequent encounter
T43642S	Poisoning by ecstasy, intentional self-harm, sequela
T43643A	Poisoning by ecstasy, assault, initial encounter
T43643D	Poisoning by ecstasy, assault, subsequent encounter
T43643S	Poisoning by ecstasy, assault, sequela
T43644A	Poisoning by ecstasy, undetermined, initial encounter
T43644D	Poisoning by ecstasy, undetermined, subsequent encounter
T43644S	Poisoning by ecstasy, undetermined, sequela
T43691A	Poisoning by other psychostimulants, accidental (unintentional), initial encounter
T43691D	Poisoning by other psychostimulants, accidental (unintentional), subsequent encounter
T43691S	Poisoning by other psychostimulants, accidental (unintentional), sequela
T43692A	Poisoning by other psychostimulants, intentional self-harm, initial encounter
T43692D	Poisoning by other psychostimulants, intentional self-harm, subsequent encounter
T43692S	Poisoning by other psychostimulants, intentional self-harm, sequela
T43693A	Poisoning by other psychostimulants, assault, initial encounter
T43693D	Poisoning by other psychostimulants, assault, subsequent encounter
T43693S	Poisoning by other psychostimulants, assault, sequela
T43694A	Poisoning by other psychostimulants, undetermined, initial encounter
T43694D	Poisoning by other psychostimulants, undetermined, subsequent encounter
T43694S	Poisoning by other psychostimulants, undetermined, sequela
T43695A	Adverse effect of other psychostimulants, initial encounter
T43695D	Adverse effect of other psychostimulants, subsequent encounter

Tobacco use

F17.xxx, Z716, Z720

© 2021 Chua KP et al. *JAMA Network Open.*

eAppendix 3. Prescriber Types

We identified combinations of primary and secondary provider taxonomy codes corresponding to the following prescriber types:

- Family medicine physicians
- Internists
- Pain medicine physicians
- Emergency medicine physicians (including pediatric emergency medicine)
- Obstetricians/gynecologists
- Physical medicine and rehabilitation physicians (excludes pain medicine)
- Surgeons (general, surgical critical care, hand, pediatric, trauma, vascular, surgical oncology, colorectal, thoracic, orthopedic, oral maxillofacial surgery, plastic surgery, neurosurgery, ophthalmology, otolaryngology, urology
- Psychiatrists (excludes pain medicine)
- Neurologists (excludes pain medicine)
- Other physicians (independent medical examiner, phlebology, neuromuscular medicine, electrodiagnostic medicine, allergy and immunology, medical genetics, nuclear medicine, pathology, pediatrics, preventive medicine, radiology, general practice, clinical pharmacology, legal medicine)
- Physician assistant
- Nurse practitioner
- Dentist
- Podiatrist
- All other providers

© 2021 Chua KP et al. *JAMA Network Open.*

eTable 3. Adjusted Association Between Overlapping Opioid And Benzodiazepine Prescriptions From Multiple Prescribers and Overdose

Exposure or Covariate	Adjusted odds ratio (95% CI)	Average marginal effect (95% CI)
Multiple prescribers accounting for overlapping opioid and benzodiazepine prescriptions (versus one)	1.20 (1.10, 1.31)	0.91 (0.46, 1.37)
Daily MME category (vs < 30)		
30-59	1.21 (1.06, 1.38)	0.85 (0.26, 1.44)
60-89	1.23 (1.05, 1.44)	0.95 (0.23, 1.66)
90-119	1.70 (1.42, 2.04)	2.87 (1.84, 3.89)
≥120	1.35 (1.13, 1.61)	1.41 (0.57, 2.26)
Daily DME category (vs <10)		
11-20	1.21 (1.06, 1.37)	0.80 (0.24, 1.36)
21-30	1.38 (1.20, 1.58)	1.46 (0.82, 2.10)
31-40	1.52 (1.30, 1.76)	2.00 (1.23, 2.78)
≥40	1.79 (1.53, 2.09)	3.08 (2.15, 4.01)
Extended-release/long-acting opioid use (vs none)	1.23 (1.07, 1.41)	1.04 (0.34, 1.74)
Mental health disorder (vs none)	1.38 (1.03, 1.85)	1.64 (0.13, 3.14)
Substance use disorder (vs none)	40.78 (34.65, 48.00)	18.83 (17.57, 20.09)
Tobacco use (versus none)	1.22 (1.10, 1.35)	0.99 (0.47, 1.52)
Cancer (vs none)	0.88 (0.78, 0.99)	-0.64 (-1.23, -0.05)
Number of Elixhauser co-morbidity flags	1.13 (1.12, 1.14)	0.62 (0.55, 0.69)
Age in single years	1.00 (1.00, 1.00)	0.00 (-0.02, 0.02)
Male (vs female)	0.74 (0.67, 0.82)	-1.45 (-1.91, -0.98)
US Census region (vs Northeast)		
Midwest	1.03 (0.83, 1.27)	0.13 (-0.92, 1.18)
South	1.02 (0.84, 1.24)	0.10 (-0.84, 1.05)
West	1.20 (0.95, 1.51)	0.96 (-0.23, 2.14)
Medicare Advantage (vs commercial insurance)	1.02 (0.90, 1.15)	0.08 (-0.54, 0.71)
Person-day occurred in 2018 (vs 2017)	0.91 (0.83, 1.00)	-0.46 (-0.93, 0.01)

eTable 4. Sensitivity Analyses

© 2021 Chua KP et al. *JAMA Network Open.*

Analysis	# person-days included in analysis	Adjusted odds ratio (95% CI)	Average marginal effect of exposure per 100,000 person-days of overlap (95% CI)
Main analysis	52,989,316	1.20 (1.10, 1.31)	0.91 (0.46, 1.37)
Exclude person-days of overlap involving prescriptions from a pain medicine physician	45,847,909	1.20 (1.09, 1.33)	0.87 (0.39, 1.35)
Excluded person-days involving any buprenorphine formulation	51,764,768	1.18 (1.08, 1.29)	0.85 (0.40, 1.31)
Excluded patients with cancer diagnosis in the 365 days prior to cohort entry	38,564,767	1.16 (1.04, 1.28)	0.72 (0.21, 1.22)
Excluded patients with substance use disorder diagnosis in the 365 days prior to cohort entry	43,416,173	1.61 (1.18, 2.18)	0.21 (0.07, 0.34)
Control for number of days since the beginning of an episode of opioid-benzodiazepine overlap	52,989,316	1.19 (1.09, 1.30)	0.88 (0.43, 1.33)
Used flexible, non-parametric thin-plate smoothing spline function rather than model daily MME and DME as categorical variables	52,989,316	1.21 (1.12-1.30)	N/A [R software package could not calculate AME when splines were used]
Did not control for daily MME, daily DME, or use of extended-release/long-acting opioids	52,989,316	1.27 (1.17, 1.39)	1.23 (0.77, 1.69)

<u>Sensitivity analysis in which exposure variable was a categorical variable for 1, 2, and ≥3 prescribers instead of 1 versus more than 1</u>

In this sensitivity analysis, covariates were the same as in the main analysis. Adjusted results for the new exposure variable are below

	AOR (95% CI)	AME (95% CI)
1 prescriber	Ref	Ref
2 prescribers	1.18 (1.08, 1.29)	0.83 (0.38, 1.29)
≥3 prescribers	1.55 (1.24, 1.96)	2.57 (0.98, 4.15)

© 2021 Chua KP et al. *JAMA Network Open.*
